# Reframing Clinical Research in Latin America: Implications of Underrepresentation for Health Systems and Global Evidence

**DOI:** 10.7759/cureus.102829

**Published:** 2026-02-02

**Authors:** Esteban Zavaleta-Monestel, Ernesto Martínez-Vargas, Jeaustin Mora-Jiménez, Sebastián Arguedas-Chacón

**Affiliations:** 1 Research Department, Hospital Clínica Bíblica, San José, CRI; 2 Pharmacy Department, Hospital Clínica Bíblica, San José, CRI

**Keywords:** clinical research capacity, global evidence equity, health system performance, latin america, oncology research, regulatory reliance, research underrepresentation

## Abstract

Although Latin America represents a substantial share of the global population and bears a significant and growing disease burden, the region remains underrepresented in global clinical research relative to its scale. This imbalance is associated with limited generation of locally relevant evidence and may influence the pace of evidence uptake, health system learning, and patient access to innovative medicines, devices, and technologies. Available analyses suggest that barriers to research participation in the region are predominantly structural, including chronic underinvestment, regulatory fragmentation, misaligned incentives, institutional constraints, and limitations related to health system scale, rather than intrinsic deficiencies in clinical or scientific capacity. Comparative regional evidence indicates that research activity varies according to governance models, regulatory alignment, and system organization, supporting the view that underrepresentation reflects modifiable system-level factors. Framing clinical research as a core health system function may strengthen healthcare performance in Latin America while enhancing the relevance, diversity, and equity of the global biomedical evidence base.

## Editorial

Clinical research is often viewed as an academic supplement to healthcare delivery, a perspective that is increasingly obsolete. In advanced health systems, research is embedded within core operations, shaping clinical norms, establishing quality standards, and enabling continuous learning through the routine generation and use of local evidence to inform guideline updates, health technology assessment, coverage decisions, and quality-improvement feedback loops. When research remains peripheral, health systems become reliant on externally generated evidence, priorities, and innovation timelines. In the context of Latin America, the central concern extends beyond participation in global trials to whether health systems are designed to generate knowledge and promote learning, rather than simply consuming external evidence [1].

Global clinical research remains geographically concentrated, with trial activity unevenly distributed across countries and income groups [1]. This concentration reflects governance decisions manifested in funding patterns, regulatory capacity, and incentives including funding priorities, regulatory design, approval processes, and institutional incentives that influence trial feasibility. Consequently, trial populations and evidence outputs are clustered in environments with minimal administrative barriers and robust research infrastructure. Analyses of clinical trial globalization have documented this uneven distribution and its implications for evidence production and the prioritization of interests [2].

Underrepresentation raises significant concerns regarding both equity and the quality of evidence. When clinical trial populations are drawn predominantly from high-income countries, external validity may be compromised, particularly when disease epidemiology, comorbidity profiles, genetic diversity, environmental exposures, and health system contexts differ across settings. Global analyses have shown that the geographic underrepresentation of low- and middle-income countries in clinical trials limits the generalizability of findings and shifts uncertainty and risk onto populations that are least represented in the evidence base, despite bearing a substantial share of the global disease burden. Persistent imbalances in trial design, conduct, and reporting further reinforce these disparities by privileging evidence generated in high-income settings while constraining the visibility and influence of research conducted in low- and middle-income regions [3-5].

Limited research participation in Latin American health systems represents a substantial performance challenge. Systems without adequate research capacity may struggle to generate locally relevant evidence necessary for guideline adaptation, health technology assessment, and coverage decisions. These limitations may contribute to the slower adoption of effective interventions, delayed discontinuation of ineffective practices, and weakened health system learning. Health systems literature emphasizes that research capacity and the ability to generate and apply evidence are fundamental components of health system performance, shaping learning, adaptation, and resource allocation [6].

Capacity-building frameworks for low- and middle-income countries recognize research capacity as a system-level asset that requires sustained investment, local ownership, and alignment with national priorities [7]. Research on minimizing waste further underscores the importance of establishing locally relevant research priorities and producing actionable evidence to maximize value and reduce inefficiency, particularly in resource-constrained settings [8].

Patient access constitutes a critical ethical consideration. In many low- and middle-income countries, clinical trials offer early access to innovative medicines, devices, and therapeutic strategies, raising significant ethical and scientific questions about globalization and inclusion [9]. Limited participation in trials may restrict access pathways and intensify inequities, affecting not only availability but also clinical familiarity and institutional readiness for new technologies. Analyses of medicine access in these settings have shown that trial participation and broader access dynamics can interact to exacerbate disparities. Normative scholarship on equitable access argues that inequity involves not only final market availability but also the distribution of research benefits and risks across diverse settings [10,11].

Comparative analyses of clinical research activity in Latin America illustrate both underrepresentation and its modifiable nature. Despite accounting for more than 8% of the global population and a growing disease burden, the region represented approximately 3.6-5% of global oncology clinical trials in 2023. Trial activity is highly concentrated, with Brazil, Mexico, and Argentina as the main contributors. Brazil alone participates in approximately 41% of phase I-III oncology trials conducted in Latin America and the Caribbean, while Mexico and Argentina contribute smaller but meaningful shares [12].

Trial volume remains modest relative to disease burden. Between 2018 and 2023, Brazil hosted approximately 1.9% of global oncology trials, Mexico 1.3%, and Argentina 1.3%, with lower participation from other countries in the region. Latin America is better represented in late-phase studies, contributing approximately 15.6% of global phase III oncology trials, but participation in early-phase research remains limited. Oncology is used here as an illustrative case because it concentrates a substantial share of global clinical trial activity and provides robust, comparable metrics across countries [12].

Regulatory approval timelines vary considerably across the region. Median trial start-up times are approximately 2.8 months in Mexico, 4.5 months in Argentina, between four and seven months in Brazil, 4.6 months in Chile, seven months in Colombia, and up to nine months in Peru. While these timelines are increasingly comparable to those in other regions, persistent heterogeneity highlights the role of regulatory alignment and governance rather than intrinsic limitations in clinical or scientific capacity [12,13].

Importantly, capacity-building initiatives, particularly during the COVID-19 pandemic, demonstrated that targeted investments can rapidly reduce approval timelines, improve site readiness, and expand trained research personnel. Taken together, these findings support the conclusion that underrepresentation in Latin America reflects modifiable system-level factors rather than fixed limitations in clinical or scientific capability [14].

Given the scale of Latin America, its underrepresentation in clinical research is particularly difficult to justify. The region is characterized by considerable demographic and socioeconomic diversity, with disease burdens encompassing both communicable and non-communicable conditions. Despite this, numerous regional assessments continue to identify deficiencies in research participation and in the infrastructure necessary to generate and use local evidence. Globally, this creates a paradox: a large and diverse region remains a minor contributor to the evidence base guiding its own clinical decisions [10].

These consequences are particularly visible in oncology, which is used here as an illustrative case given its high volume of global clinical trial activity, rapid innovation cycles, and the availability of comparable international metrics. Regional analyses of cancer research and control in Latin America have identified both progress and persistent gaps, including the influence of research ecosystems on care pathways and the uptake of innovations [15]. Further studies indicate that disparities in research participation in oncology are associated with differences in evidence generation and may influence access pathways and clinical readiness for new therapies, without implying uniform outcome effects across the region [16].

It is important to recognize that Latin America is not a homogeneous research environment. Brazil, for instance, has established extensive research networks and institutional capacity, supporting sustained participation in clinical trials and broader research activities. This example illustrates that underrepresentation is not inevitable. It also underscores a key policy insight: research capacity is built through long-term commitments, including stable investment, talent retention, and regulatory systems that support predictable participation [17].

Mid-capacity systems such as Argentina, Mexico, and Chile exhibit increasing participation and academic strength; however, scaling is limited when approval processes are inconsistent, coordination is fragmented, and institutional procedures are not designed for large-scale research operations. Comparative analyses of the Latin American regulatory landscape demonstrate that differences in regulatory structure and alignment influence both drug registration and clinical research. The central editorial argument extends beyond calls to streamline regulation: predictability is not merely a bureaucratic convenience but a critical system indicator. It signals to sponsors and investigators whether the system can support a sustained research pipeline rather than only isolated studies [18].

Smaller health systems face distinct challenges. For example, Costa Rica and parts of Central America demonstrate that strong clinical care and universal coverage do not necessarily translate into competitiveness in clinical trials. Limited scale constrains recruitment, and centralized governance can hinder the contracting agility and operational flexibility required for sponsor-driven trials. These challenges reflect a mismatch in system design rather than a moral shortcoming. The definition of research success is therefore critical. If small systems equate research success solely with large sponsor-driven randomized trials, they are likely to underperform. However, if success encompasses pragmatic trials, registry-based studies, and implementation research, smaller systems can produce locally actionable evidence and enhance health system learning. The broader evidence literature has emphasized that health decision-making often requires approaches beyond traditional randomized controlled trials, particularly for evaluating policies and system-level interventions [19].

Investment remains a persistent constraint. Latin American countries generally allocate less funding to research and development than high-income nations, which limits infrastructure, career development, and the capacity for investigator-driven research aligned with local priorities. Global data consistently indicate low research and development expenditure across many countries in the region, and Organisation for Economic Co-operation and Development (OECD) indicators similarly reveal structural gaps in research investment capacity. Sustained competitiveness in clinical trials requires long-term investment, analogous to the necessity of adequate funding for excellence in tertiary care [20,21].

Regulation constitutes another persistent constraint, though the challenge extends beyond regulatory speed. In resource-limited settings, strategies such as regulatory reliance and complementarity allow authorities to minimize duplication, focus limited capacity on context-specific decisions, and utilize trusted external assessments without sacrificing rigor. Research on regulatory reliance demonstrates its potential to enhance efficiency while maintaining oversight [22]. Empirical studies further show that collaborative regulatory pathways can enable more timely action, a strategy particularly relevant during public health emergencies. For Latin America, a central policy question is whether reliance mechanisms will be used to build sustainable regulatory capacity or whether ongoing fragmentation will persist as an unaddressed operational cost [23].

Regional initiatives to build trial capacity during the COVID-19 pandemic illustrated that targeted readiness efforts can expand participation [14]. Broader recommendations to strengthen clinical trial capacity in resource-limited settings emphasize that such capacity can be developed if systems treat it as core infrastructure rather than temporary project work. The experience during periods of high urgency demonstrates that many constraints are a matter of priority and design, rather than inevitability [24].

Reframing clinical research in Latin America should not focus solely on increasing the number of trials. Instead, it requires recognizing evidence generation as a core health system function that shapes equity, resilience, and self-reliance. The region does not require a uniform approach. Large hubs require sustainability and governance that maintains trust; mid-capacity systems require predictability and opportunities for scaling; smaller systems require research models tailored to their scale and that incentivize the production of locally valuable evidence. The ongoing cost of underrepresentation is not merely a missed opportunity; it represents a subtle form of dependency that delays innovation, impedes learning, and limits the diversity reflected in global evidence.
